# Prevalence of alcohol consumption and its risk factors among university students: A cross-sectional study across six universities in Myanmar

**DOI:** 10.1371/journal.pone.0229329

**Published:** 2020-02-21

**Authors:** Hein Htet, Yu Mon Saw, Thu Nandar Saw, Nang Mie Mie Htun, Khaing Lay Mon, Su Myat Cho, Thinzar Thike, Aye Thazin Khine, Tetsuyoshi Kariya, Eiko Yamamoto, Nobuyuki Hamajima

**Affiliations:** 1 Department of Preventive and Social Medicine, University of Medicine, Mandalay, Myanmar; 2 Department of Healthcare Administration, Nagoya University Graduate School of Medicine, Nagoya, Japan; 3 Nagoya University Asian Satellite Campuses Institute, Nagoya, Japan; 4 Department of Community and Global Health, Graduate School of Medicine, the University of Tokyo, Tokyo, Japan; 5 Myanma Perfect Research, Yangon, Myanmar; 6 Department of Health Behaviour and Communication, University of Public Health, Yangon, Myanmar; 7 Department of Food and Drug Administration, Ministry of Health and Sports, Nay Pyi Taw, Myanmar; 8 Department of Public Health, Ministry of Health and Sports, Nay Pyi Taw, Myanmar; University of Ghana, GHANA

## Abstract

**Background:**

Globally, alcohol consumption is a significant public health concern and it is one of the most important risk behaviours among university students. Alcohol consumption can lead to poor academic performance, injuries, fights, use of other substances, and risky sexual behaviours among students. However, the study explored the prevalence of alcohol consumption and the associated risk factors among university students since these have not been fully examined in previous research. Therefore, the aim of this study was to explore the prevalence of alcohol consumption and the associated risk factors among university students in Myanmar.

**Methods:**

The present cross-sectional study was conducted using a sample of 15-24-year-old university students who were selected from six universities in Mandalay, Myanmar, in August 2018. In total, 3,456 students (males: 1,301 and females: 2,155) were recruited and asked to respond to a self-administered questionnaire. Multiple logistic regression analysis was used to estimate the adjusted odds ratio (AOR) and 95% confidence interval (CI) for alcohol consumption among university students.

**Results:**

The prevalence of alcohol consumption in the previous 30 days was 20.3% (males: 36.0%, females: 10.8%). The alcohol consumption was significantly higher among males (AOR = 2.3, 95% CI; 1.9–2.9), truant students (AOR = 2.1, 95% CI; 1.3–3.3), smokers (AOR = 7.0, 95% CI; 5.1–9.7), students who reported feeling of hopelessness or sadness (AOR = 1.4, 95% CI; 1.2–1.8), peers’ alcohol consumption (AOR = 7.5, 95% CI; 4.8–11.7).

**Conclusion:**

The present study revealed that males, smokers, peer alcohol consumption, and truant students had higher odds of alcohol consumption among the students. Therefore, effective campus-based counselling, peer education, and national surveillance systems that can monitor risky drinking behaviours among university students should be implemented. Further, government regulations that control the production, sale, promotion, advertising, and restriction of alcohol should be well developed and strengthened, as in the case of other Southeast Asian countries.

## Introduction

Globally, alcohol consumption is a significant public health concern and it is one of the most important risk behaviours among young adults, including university students. Currently, 26.5% of the global population between the ages of 15 and 19 consumes alcohol [1]. In the United States, the 2017 Youth Risk Behaviour Survey reported that 29.8% of their student participants had consumed alcohol in the previous 30 days [2]. In Europe, the prevalence of alcohol use among university students was reported as follows: 46.2% (males) and 28.1% (females) in Bulgaria, 41.1% (males) and 18.1% (females) in Germany, and 20.1% (males) and 10% (females) in Poland, respectively [3]. Further, Korean and Filipino college students reported higher levels of alcohol consumption than their Chinese and Vietnamese counterparts [4]. In Japan, approximately 56.8% and 47.8% of male and female university students were binge drinkers [5].

In Southeast Asia, the prevalence of harmful alcohol use among university students is 24.4% in Laos, 10.8% in Thailand, 1.4% in Myanmar, and 0.7% in Indonesia [6]. The World Health Organization (WHO) recently reported that the prevalence of heavy episodic drinking among those between the ages of 15 and 19 is higher among males (47.2%) than among females (16.1%) in Myanmar [1].

Alcohol consumption accounts for 5.9% of annual global deaths, a significant proportion of which occur among youth [1]. An Asian multi-country study, which included Japan, reported that the alcohol-related mortality rate among 15 to 24-year-old youth was 15% among males and 6% among females [7]. Previous studies have reported that alcohol consumption can lead to poor academic performance, injuries, fights, the use of substances, and risky sexual behaviours among youth [8,9].

Moreover, alcohol consumption is an important modifiable risk factor for many non-communicable diseases (NCDs) [10]. Therefore, the reduction of harmful alcohol consumption has been specifically integrated into the Global Action Plan for the Prevention and Control of NCDs 2013–2020 and Sustainable Development Goals: Goal 3, target 3.5 [11]. However, at the international level, alcohol remains the only psychoactive substance that is not controlled by legally binding regulatory frameworks [11]. Similarly, in most Asian countries including Myanmar, alcohol consumption in youth and early adulthood is ascribed relatively low importance in the national health agenda [12].

Myanmar is one of the developing countries in Southeast Asia, and it has no current national legal policy regulating alcohol consumption among youth. Additionally, it does not have a robust national surveillance system to monitor risky alcohol consumption among youths. However, the WHO reported that 15,953 people had died from alcohol-related problems such as liver cirrhosis, road traffic injuries and cancers in 2016 in Myanmar [1]. Young people constitute 28% (16 million) of the total population in Myanmar. Nevertheless, youth health indicators have not been included in the National Health Management Information System in Myanmar [13]. Moreover, the risk behaviours among youth, including university students, have not been fully examined and information about alcohol consumption and its associated risk factors among 15 to 24-year-old university students in Myanmar is scant.

Studies on alcohol consumption conducted in Southeast Asian countries, including Myanmar, have primarily focused on school age students; consequently, there is little information about university students. University life is a major developmental transition period commonly associated with addictive behaviours (such as alcohol consumption) that can be intensified by the college environment and may lead to dependency and abuse during college years [4]. Given its serious implications for future health (e.g., heavy episodic drinking, violence, social isolation), alcohol consumption among university students remains an important area of research in Myanmar.

Therefore, the present study aimed to examine the prevalence of alcohol consumption and its associated risk factors among students. The findings are expected to inform university students about the possible dangers of alcohol consumption, and to contribute significantly to national guidelines, targets, policies, and strategies that pertain to the management and evaluation of youth health programs in Myanmar.

## Methods

### Study area and participants

A cross-sectional study was conducted across six universities in Mandalay, the Republic Union of Myanmar, in August 2018. Undergraduates studying arts and sciences, and healthcare related discipline participated in this study. The inclusion criteria were: 1) undergraduates aged 15–24, 2) attending university during the 2018–2019 academic year, 3) having no apparent physical or psychological disability (students diagnosed with mental disorders or under treatment were excluded), and 4) having consented to participate. A further eligibility inclusion criterion was their presence at the institutions at the day and time of data collection. In total, 3,456 university students between the ages of 15 and 24 were recruited. The data were collected during a one-month period and separately at each university on different days (about one to two classes per day at each university).

### Sampling procedure

This study applied a two-stage probability sampling design that counted each university and classes with primary and secondary sampling units to recruit the representative samples. In the first stage, universities were selected on the bases of probability, proportional to the size of enrolment, and permission from representative authorities. In total, six out of seventeen universities were selected. In the second stage, a random sampling method was used to select the classrooms from each grade (with a random first pick) within the universities included in the first stage. This sampling offered an equal chance for each student to be chosen. All classrooms in each selected university were included in sampling frame. All students in the selected classes were invited to participate in this study.

### Measure and data collection

The data were collected during a 45-minute class period using a pre-tested, self-administered questionnaire. It was adopted from the 2017 National Youth Risk Behavioural Surveillance Survey [2] and the 2016 Myanmar Global School-based Student Health Survey (GSHS) [11] that were developed by the WHO, the Centers for Disease Control and Prevention (CDC), and the Ministry of Health and Sports, Myanmar. The original English GSHS was translated, modified, and validated into Myanmar language by the Myanmar Ministry of Health and Sports. This study relied on the Myanmar language version of GSHS. The pre-test also was conducted among university students outside Mandalay Regions. The internal consistency (Cronbach’s alpha) of the pre-test and post-test was more than 0.80. The questionnaire consists of the items that require information about demographic characteristics and assess the important risk behaviours of students, including alcohol consumption.

### Dependent variable

The main outcome measure (dependent variable) was “alcohol consumption” among university students. It was defined as “consuming at least one drink of alcohol (includes drinking a bottle of beer, a glass of wine, a glass of liquor such as whisky, rum, cocktail or mixed drink) on at least one day in the 30 days prior to the survey, but excludes drinking a few sips of wine for religious purposes”. The responses to this question were 0 days, 1–2 days, 3–5 days, 6–9 days, 10–19 days, 20–29 days, and all 30 days. It was then recoded as “no day (0)” and “1 or more days (1)” on a dichotomous scale.

### Independent variables

The independent variables used for multiple logistic regression analysis were age (over or under 18), sex (female or male), monthly expenses (<120,000 Myanmar kyats or ≥120,000), academic year (first, second, third, fourth, fifth, or above), ethnicity (Bamar or others), living status (with family/relatives or alone), parents’ or guardians’ alcohol consumption (no or yes), peer alcohol consumption (no and yes), unauthorized absence from class in the previous 30 days (no or yes), smoking in the previous 30 days (no or yes), smokeless tobacco use in the previous 30 days (no or yes), ever use illicit drug (no or yes), ever attempted suicide in the previous 12 months (no or yes), being supervised by parents or guardians (no or yes), and hopelessness or sadness in the previous 12 months (no or yes).

### Statistical analysis

The data analysis was conducted using the Statistical Package for Social Science (SPSS) software program version 24.0. Descriptive statistics (frequency and percentage) were computed for socio-demographics, the behavioural characteristics related to alcohol consumption and the associated risk factors. To examine risk factors, a multiple logistic regression analysis was conducted and the adjusted odds ratio (AOR) and 95% confidence interval (CI) were calculated. Using the forward method, age, sex, and monthly expenses were included in each step of the logistic regression model. Analysis was two-tailed, with a p-value of <0.05 considered to be statistically significant. As instructed by universities authorities, university names were not recorded.

### Ethical considerations

This study was approved by the Institutional Technical and Ethical Review Board of the University of Public Health, Yangon, Myanmar (approval letter no. UPH-IRB 2018/Research/26 issued on the 31st of July 2018). Both verbal and written informed consents were obtained from students after explaining the aims, the procedure, possible benefits of the study, contents of the questionnaires, and the individual right to withdraw from the study without penalty. Moreover, the study information sheet that included study objectives, the contents of questionnaires, and rights of study participants were distributed to students, guardians, and parents one week prior to survey start date. This study recruited some minor students without their parents’ or guardians’ consent due to living away from their parents to study at University in Mandalay city area. Many Myanmar students start living independently when they enroll to University. The Institutional Technical and Ethical Review Board waived to obtain the parental and guardian informed consents and approved interview procedures due to the nature of study participants. However, the permission to conduct the university-based survey, and consent from local university academic and administrative board members needed to be obtained. In addition, the permission to conduct the university-based survey including the verbal and written consents were obtained from academic and administrative board members (Rectors, Pro-rectors, Registrars, Professors, and Heads of the teaching departments). The study was designed to entail anonymous and voluntary participation to protect the privacy of the students. Hence, students’ name and universities were not recorded. The data were kept confidential throughout all stages of data collection and analysis.

## Results

### Descriptive statistics

#### Background characteristics of university students

The sample (N = 3,456) consisted of 1,301 boys (37.6%) and 2,155 girls (62.4%) representing six universities. Their mean age was 18.7 years (SD = 1.6). More than half of the male (56.2%) and female (61.4%) participants were between the ages of 18 and 20. Approximately three-quarters of the sample belonged to the Bamar (74.0%) ethnic group. Further, approximately half of the males (49.5%) and more than half of the females (62.2%) lived with their family members. The monthly expenditure of more than 65.0% of males and females ranged from 100,000 to 300,000 kyats (Table 1).

**Table 1:** Background characteristics of university students (N = 3,456).

Characteristics	Male (n = 1,301)		Female (n = 2,155)		Total (N = 3,456)	
	n	%	n	%	N	%
**Age group (years)**						
15–17	296	22.8	599	27.8	895	25.9
18–20	731	56.2	1,324	61.4	2,055	59.5
21–24	274	21.0	232	10.8	506	14.7
**Academic year**						
First year	328	25.2	601	27.9	929	26.9
Second year	344	26.4	478	22.2	822	23.8
Third year	267	20.5	544	25.2	811	23.5
Fourth year	187	14.4	285	13.2	472	13.7
Fifth and above	175	13.5	247	11.5	422	12.2
**Ethnicity**						
Kachin	39	3.0	34	1.6	73	2.1
Kayar	3	0.2	6	0.3	9	0.3
Kayin	6	0.5	20	0.9	26	0.8
Chin	15	1.2	21	1.0	36	1.0
Bamar	947	72.8	1,611	74.8	2,558	74.0
Mon	15	1.2	15	0.7	30	0.9
Rakhine	7	0.5	11	0.5	18	0.5
Shan	103	7.9	142	6.6	245	7.1
Others*	166	12.8	295	13.7	461	13.3
**Living status**						
With family/relatives	644	49.5	1,383	64.2	2,027	58.7
Alone	657	50.5	772	35.8	1,429	41.3
**Monthly expense (MMK**^#^**)**						
Less than 100,000	342	26.3	673	31.2	1,015	29.4
100,000 to 300,000	897	68.9	1,415	65.7	2,312	66.9
300,001 and more	62	4.8	67	3.1	129	3.7

*Indians, Chinese, and multi-ethnics,

^#^Myanmar kyat (1USD = 1520.90 MMK on 19th April, 2019)

#### Smoking, smokeless tobacco, drug use and other characteristics among university students

Table 2 shows that 22.2% of males and 1.2% of females had used some form of smoking tobacco at least once in the 30 days prior to the survey; similarly, 36.0% of males and 10.8% of females had made use of smokeless tobacco. Moreover, 2.5% of the students reported lifetime substance use (e.g., marijuana, amphetamines, inhalants); the sex-specific frequencies were 6.0% for males and 0.4% for females. Approximately one-third of both males (29.8%) and females (34.3%) reported feeling sad or hopeless in the 12 months preceding the survey, and approximately ten percent (9.7%) reported that they had attempted suicide during this time period. The majority of participants (94.3%) reported that they had not been monitored by their parents or guardians in the previous 30 days. Additionally, almost all students had a history of missing class without the requisite permission in the 30 days prior to the survey (males: 89.9%, females: 86.8%).

**Table 2:** Frequency of smoking, smokeless tobacco use, drug use and other characteristics among university students (N = 3,456).

Characteristics	Male (n = 1,301)		Female (n = 2,155)		Total (N = 3,456)	
	n	%	n	%	N	%
**Smoking in the previous 30 days**						
No	1,012	77.8	2,129	98.8	3,141	90.9
Yes	289	22.2	26	1.2	315	9.1
**Smokeless tobacco uses in the previous 30 days**						
No	833	64.0	1,922	89.2	2,755	79.7
Yes	468	36.0	233	10.8	701	20.3
**Ever use illicit drug**						
No	1,223	94.0	2,147	99.6	3,370	97.5
Yes	78	6.0	8	0.4	86	2.5
**Feeling sadness or hopeless in the previous 12 months**						
No	914	70.3	1,415	65.7	2,329	67.4
Yes	387	29.8	740	34.3	1,127	32.6
**Ever attempt suicide in the previous 12 months**						
No	1,182	90.9	1,939	90.0	3,121	90.3
Yes	119	9.1	216	10.0	335	9.7
**Being supervised by parents (or) guardians**						
No	1,202	92.4	2,056	95.4	3,258	94.3
Yes	99	7.6	99	4.6	198	5.7
**Absence of class without permission in the previous 30 days**						
No	131	10.1	284	13.2	415	12.0
Yes	1,170	89.9	1,871	86.8	3,041	88.0

#### Characteristics related to alcohol consumption among university students

Table 3 presents the findings that are related to students’ alcohol consumption. Overall, the prevalence of alcohol consumption was 20.3% (males: 36.0%, females: 10.8%). More than half of the males (52.8%) and approximately one-fourth (24.8%) of the females started drinking at the age of 10. Further, more than one-third of both males (38.0%) and females (36.0%) reported parental alcohol consumption, and almost all males (88.1%) and more than half of females (60.4%) reported peer alcohol consumption. Approximately 10% of the students had encountered social conflicts or problems as a consequence of drinking.

**Table 3:** Characteristics related to alcohol consumption among university students (N = 3,456).

Characteristics	Male (n = 1,301)		Female (n = 2,155)		Total (N = 3,456)	
	n	%	n	%	N	%
**Age of alcohol initiation**						
Never or non-drinker	601	46.2	1,604	74.4	2,205	63.8
Less than 10 years	13	1.0	17	0.8	30	0.9
10 years or more	687	52.8	534	24.8	1,221	35.3
**Alcohol consumption in the previous 30 days**						
No	833	64.0	1,922	89.2	2,755	79.7
Yes	468	36.0	233	10.8	701	20.3
**Frequency of alcohol consumption in the previous 30 days**						
Never or non-drinker	833	64.0	1,922	89.2	2,755	79.7
1–9 days	407	31.3	229	10.6	636	18.4
10 days and above	61	4.7	4	0.2	65	1.9
**Source of getting alcohol**						
Never or non-drinker	833	64.0	1,922	89.2	2,755	79.7
Bought in a store or shop	283	21.8	117	5.4	400	11.6
from friends or family	113	8.7	77	3.6	190	5.5
Got some other way	72	5.5	39	1.8	111	3.2
**Parents’ or guardians’ alcohol consumption**						
No	807	62.0	1,379	64.0	2,186	63.2
Yes	494	38.0	776	36.0	1,270	36.8
**Peers’ alcohol consumption**						
No	155	11.9	853	39.6	1,008	29.2
Yes	1,146	88.1	1,302	60.4	2,448	70.8
**Social problems due to alcohol in the previous 30 days**						
No	1,101	84.6	2,014	93.5	3,115	90.1
Yes	200	15.4	141	6.5	341	9.9

#### Multivariate analysis was used to explore the risk factors for alcohol consumption among university students

Table 4 shows the results of the logistic regression analysis that pertained to alcohol consumption. The odds of consumption were high among males (AOR = 2.3, 95% CI; 1.9–2.9), smokers (AOR = 7.0, 95% CI; 5.1–9.7), older students (AOR = 1.5, 95% CI; 1.2–1.8), truant students (AOR = 2.1, 95% CI; 1.3–3.3), those whose monthly expenses were greater than or equal to 120,000 kyats (AOR = 1.8, 95% CI; 1.5–2.3), and those who reported feelings of sadness or hopelessness (AOR = 1.4, 95% CI; 1.2–1.8). Importantly, alcohol consumption by parents or guardians (AOR = 1.4, 95% CI; 1.2–1.8) and peers (AOR = 7.5, 95% CI; 4.8–11.7) increased the odds of alcohol consumption among students.

**Table 4:** Odds ratio (OR) and 95% confidence interval (CI) of alcohol consumption in the previous 30 days among university students (N = 3,456).

Characteristics	Alcohol consumption (n = 701)		No alcohol consumption (n = 2,755)		Unadjusted		Adjusted ^a)^	
	n	%	n	%	OR	95% CI	OR	95% CI
**Sex**								
Female	233	33.2	1,922	69.8	1	Reference	1	Reference
Male	468	66.8	833	30.2	4.6	(3.9–5.3)***	2.3	(1.9–2.9) ***
**Age (in years)**								
Under 18	103	14.7	792	28.8	1	Reference	1	Reference
18 or older	598	85.3	1,963	71.3	2.3	(1.9–2.9) ***	1.5	(1.2–1.8) **
**Monthly expenses (MMK**^#^**)**								
<120,000	218	31.1	1,472	53.4	1	Reference	1	Reference
≥120,000	483	68.9	1,283	46.6	2.5	(2.1–3.0) ***	1.8	(1.5–2.3) ***
**Parents’ or guardians’ alcohol consumption**								
No	374	53.4	1,812	65.8	1	Reference	1	Reference
Yes	327	46.7	943	34.2	1.7	(1.4–2.0) ***	1.4	(1.2–1.8) ***
**Peers’ alcohol consumption**								
No	22	3.1	986	35.8	1	Reference	1	Reference
Yes	679	96.9	1,769	64.2	17.2	(11.2–26.5) ***	7.5	(4.8-11.7)***
**Absence of class without permission in the previous 30 days**								
No	26	3.7	389	14.1	1	Reference	1	Reference
Yes	675	96.3	2,366	85.9	4.3	(2.8–6.4) ***	2.1	(1.3–3.3) **
**Smoking in the previous 30 days**								
No	456	65.1	2,685	97.5	1	Reference	1	Reference
Yes	245	35.0	70	2.5	20.6	(15.5–27.4) ***	7.0	(5.1–9.7) ***
**Feeling sadness or hopeless in the previous 12 months**								
No	423	60.3	1,906	69.2	1	Reference	1	Reference
Yes	278	39.7	849	30.8	1.5	(1.2–1.8) ***	1.4	(1.2–1.8) ***

^#^Myanmar kyat (1USD = 1520.90 MMK on 19^th^ April, 2019)

^a)^ Adjusted for sex, age group, academic year, monthly expenses, parents’ or guardians’ alcohol consumption, friends’ alcohol consumption, absence of class without permission, smoking, feeling sadness or hopelessness in a forward method, compulsorily including sex, age group, academic year, and monthly expenses.

*P<0.05,

**P<0.01,

***P<0.001

## Discussion

The present study revealed that sex, age, monthly expenses, parental and peer alcohol consumption, smoking habits, truancy, and feelings of sadness or hopelessness were positively associated with alcohol consumption. The prevalence of alcohol consumption among 15 to 24-year-old university students (i.e. in the 30 days prior to survey) was 20.3%. This figure is four times higher than 4.5% [11] which is the prevalence rate that was reported by the 2016 Myanmar Nationwide GSHS conducted among youth between the ages of 13 and 17. The higher prevalence rate found in this study may be attributable to the following factors: coping with the new university environment, living away from one’s family, lower levels of parental monitoring, and peer pressure. These factors may cause university students to engage in substance abuse behaviours to a greater extent than school-age youth [14]. A Serbian study found that university students consume alcohol to relieve stress and improve mood [15]. Moreover, alcohol was found to be the substance that was most abused by university students in the United Kingdom and Ireland [16].

However, the reported prevalence of alcohol consumption is lower in our study than in those conducted in Asia [17–19], Europe [20–22] and African region [23, 24]. This may be attributable to geographical differences in beliefs, religious and cultural practices, environmental factors, the accessibility and availability of alcohol, urbanization, lifestyle, national laws, and governmental and legal enforcement of alcohol-related regulations [1].

The present findings show that students who had missed classes without permission in the 30 days prior to the study demonstrated a more-than-twofold increase in alcohol consumption. This trend is consistent with previous findings that truant students are more likely to report alcohol consumption and alcohol-related problems than non-truant students [25–27]. Truancy is linked to an unfavourable educational environment and physical or mental disability among students. This suggests that truant students require greater levels of attention, supervision, and support. Accordingly, the WHO recommends the promotion of positive attitudes and positive student-teacher relationships as a means to protect students from substance abuse and depression [2]. Therefore, school authorities must create supportive university environments that enhance the physical, mental, and social well-being of students. Campus-wide interventions, web-based interventions and brief motivational interventions targeting addictive behaviours among university students have proved effective [28, 29].

In this study, students who felt hopeless or sad were more likely to consume alcohol than their hopeful and happy counterparts. Similar findings were reported in previous studies [30–34]. Hopelessness is highly relevant condition that could motivate someone to consume alcohol [30]. Similarly, substance use is closely related to factors such as hopelessness and anxiety sensitivity [35]. People who experience feelings of hopelessness tend to harbour negative expectations about future life events and are more likely to experience depressive disorders or depend on substances like benzodiazepines, opiates, and alcohol [36]. Academic stress, the pressure to succeed, and peer competition may cause university students to relieve their stress by consuming alcohol [37]. Therefore, counselling centres in universities must be more proactive and help students who exhibit the symptoms of depression or experience feelings of hopelessness. Intervention strategies that alleviate these feelings may be effective in preventing alcohol consumption among university students. In Myanmar, a mental health project was launched in 2006 and integrated into the school health services. However, there are several challenges in promoting the mental health of students due to an inadequate mental health workforce [11]. Therefore, effective institutional policies that facilitate the prevention of and education about the potentially negative psychological states of students (e.g. hopelessness) are required.

Our study showed that students who smoked in the previous 30 days before the study were seven times more likely to consume alcohol than their non-smoking counterparts. Cigarette smoking during youth is strongly linked to subsequent alcohol use and abuse [6,20,37–40]. A systematic review also concluded that a higher quantity and frequency of alcohol consumption was associated with a higher prevalence of tobacco smoking among European university students [41]. In Myanmar, substance use and its related disorders are significant problems that affect youth [13]. In addition, students have easy access to alcoholic beverages in close proximity to their university because there is no legal regulation in Myanmar that controls the sale or promotion of alcohol within the areas surrounding an educational institution [3]. In Thailand, the Alcohol Control Act prohibits the sale of alcoholic beverages within 300 metres of higher educational institutions [9]. Myanmar needs similar robust alcohol-control policies that restrict the sale and promotion of alcohol beverages around educational institutions and sponsorship of sports or youth events in university settings.

In the present study, one of the strongest predictors of alcohol consumption is peer alcohol consumption. Indeed, students with friends who consumed alcohol were seven times more likely to consume alcohol than those without. This finding concurs with the results of other studies [42,43]. For example, a study that was conducted in Thailand showed that peer influence has a significant impact on the initiation to alcohol consumption among youth [17]. Many students consider this to be normative and acceptable behaviour and a key social channel [44]. In recent years, alcohol consumption has become a popular social lifestyle behaviour among students in Myanmar. Therefore, campus-based counselling programs and harm-reduction strategies that screen for alcohol misuse and address students’ perceptions of peer alcohol consumption should be developed. Moreover, awareness campaigns that rely on media and peer education should be developed to facilitate positive behavioural changes among university students.

Parents’ or guardians’ alcohol consumption emerged as a significant risk factor in the present study. In Myanmar, family is extremely important. Parents and guardians serve as role models for children, and exert great influence on adolescents’ drinking behaviour. Two studies that were conducted in Norway [45] and Ireland [46] showed that adolescents whose fathers misused alcohol consumed more alcohol than their counterparts of the same sex and age, whose fathers did not misuse alcohol. A systematic review also concluded that parental modelling is positively associated with adolescents’ early alcohol consumption [47].

Unsurprisingly, male students were twice as likely to consume alcohol than the female students in this study. This may be due to the majority of people in Myanmar being Theravada Buddhists, and intoxication through alcohol, drugs and other means is prohibited in their daily life [48]. Due to the influence of Buddhism, Myanmar society considers smoking, drug abuse, and alcohol drinking as a bad behaviour. However, alcohol (especially homemade alcohol and palm tree juice) is served at traditional and/or religious events in some Myanmar ethnic groups. Although society considers alcohol drinking as unacceptable behaviour for both males and females, disapproval is greater for the latter, while being regarded as one of the “wild behaviours” of males [49]. Therefore, traditionally, Myanmar women do not often drink alcohol and men consider it a common health-risk behaviour. This result confirmed previous study on substance abuse in Myanmar, which found that gender was a key risk factor for the early initiation of substance use [50]. Moreover, the global trends of alcohol consumption also highlighted that males often consumed higher amounts of alcohol and were more likely to be alcohol dependent than females [1,9,20,51,52]. Across all the WHO regions (including Myanmar), fewer females reported alcohol consumption than males [1]. This finding concurs with sex differences in the prevalence of alcohol use disorder (males: 3.2%, females: 0.6%) and alcohol dependence (males: 1.3%, females: 0.2%) in Myanmar [1]. Therefore, gender-specific alcohol prevention programs are urgently needed for the benefit of university students in Myanmar.

This study provides the most current prevalence of alcohol consumption and its associated risk factors among university students in Myanmar. However, these findings must be interpreted within the confines of several limitations. First, the collected data, including alcohol consumption, was measured using a self-reported questionnaire; however, we ensured anonymity to reduce subjective bias. Nevertheless, self-report measures are important means of data collection and key sources of information, particularly in Southeast Asian countries where there is a scarcity of data on health-risk behaviours among youth. Second, the cross-sectional design of the present study limits the establishment of causal relationships between sociodemographic and behavioural factors, and alcohol consumption. Third, this study included university students in Mandalay city who were present on the day of the survey; this limits the generalistion of the findings to other demographic groups. Further, students who were absent or suspended from school may have been more likely to consume alcohol. If this is indeed the case, the overall prevalence of alcohol consumption that emerged in the present study might be an underestimate. However, given the large sample size (N = 3,456) of the study, the results may be considered to be robust and representative of the corresponding population.

### Conclusion

In conclusion, the prevalence of alcohol consumption among 15 to 24-year-old university students in Mandalay, Myanmar, was 20.3% (males: 36.0%; females: 10.8%). Age, sex, monthly expenses, parental alcohol consumption, peer alcohol consumption, truancy, and feelings of sadness or hopelessness were risk factors for alcohol consumption. Therefore, effective campus-based counselling, peer education, and national surveillance systems that can monitor risky drinking behaviours among youth should be developed and implemented. Furthermore, governmental regulations regarding the production, sale, promotion, advertising, and restriction of alcohol should be well developed and strengthened in a manner that is similar to what is done in other Southeast Asian countries.

## References

[1] World Health Organization. Global status report on alcohol and health 2018. Geneva: World Health Organization 2018.

[2] Kann L, McManus T, Harris WA, Shanklin SL, Flint KH, Queen B, et al Youth Risk Behavior Surveillance—United States, 2017. MMWR Surveill Summ. 2018;67(8):1–114. 10.15585/mmwr.ss6708a1 29902162 PMC6002027

[3] Mikolajczyk RT, Sebena R, Warich J, Naydenova V, Dudziak U, Orosova O. Alcohol drinking in university students matters for their self-rated health status: A Cross-sectional study in three European countries. Front Public Health. 2016;4:210 10.3389/fpubh.2016.00210 . 27730122 PMC5037320

[4] Lum C, Corliss HL, Mays VM, Cochran SD, Lui CK. Differences in the drinking behaviors of Chinese, Filipino, Korean, and Vietnamese college students. J Stud Alcohol Drugs [Internet]. 2009 7 [cited 2019 Jan 8];70(4):568–574. https://www.ncbi.nlm.nih.gov/pmc/articles/PMC2696297 10.15288/jsad.2009.70.568 19515297 PMC2696297

[5] Yoshimoto H, Takayashiki A, Goto R, Saito G, Kawaida K, Hieda R, et al Association between excessive alcohol use and alcohol-related injuries in college students: A multi-center cross-sectional study in Japan. Tohoku J Exp Med. 2017;242(2):157–63. https://doi-org.ejgw.nul.nagoya-u.ac.jp/10.1620/tjem.242.15 28637993 10.1620/tjem.242.157

[6] Yi S, Ngin C, Peltzer K, Pengpid S. Health and behavioral factors associated with binge drinking among university students in nine ASEAN countries. Subst Abuse Treat Prev Policy. 2017;12(1):32 10.1186/s13011-017-0117-2 . 28651601 PMC5485584

[7] Jiang H, Xiang X, Hao W, Room R, Zhang X, Wang X. Measuring and preventing alcohol use and related harm among young people in Asian countries: a thematic review. Glob Health Res Policy. 2018;3:14 10.1186/s41256-018-0070-2 . 29761160 PMC5941657

[8] Manickam MA, Abdul Mutalip MHB, Abdul Hamid HAB, Kamaruddin RB, Sabtu MYB. Prevalence, comorbidities, and cofactors associated with alcohol consumption among school-going adolescents in Malaysia. Asia Pac J Public Health. 2014;26(5 Suppl):91S–9S. 10.1177/1010539514542194 25038196

[9] So ES, Park BM. Health Behaviors and Academic Performance Among Korean Adolescents. Asian Nurs Res. 2016;10(2):123–7. 10.1016/j.anr.2016.01.004. 27349669

[10] World Health Organization. Global action plan for the prevention and control of noncommunicable diseases 2013–2020. Geneva: World Health Organization 2013.

[11] World Health Organization, Regional Office for South-East Asia and Ministry of Health and Sports, Republic of the Union of Myanmar. Report of the second Global School-based Student Health Survey (2016) in Myanmar. New Delhi: World Health Organization 2018.

[12] Casswell S, Thamarangsi T. Reducing harm from alcohol: call to action. Lancet. 2009;373(9682):2247–57. 10.1016/S0140-6736(09)60745-5 19560606

[13] Ministry of Health, Myanmar. Five-Year Strategic Plan for Reproductive (2014–2018). Department of Public Health, Ministry of Health, Myanmar. 2014.

[14] Davoren MP, Shiely F, Byrne M, Perry IJ. Hazardous alcohol consumption among university students in Ireland: a cross-sectional study. BMJ Open. 2015;5(1):e006045 10.1136/bmjopen-2014-006045 . 25633284 PMC4316479

[15] Tosevski DL, Milovancevic MP, Gajic SD. Personality and psychopathology of university students. Curr Opin Psychiatry. 2010;23(1):48–52. 10.1097/YCO.0b013e328333d625 . 19890212

[16] Davoren MP, Demant J, Shiely F, Perry IJ. Alcohol consumption among university students in Ireland and the United Kingdom from 2002 to 2014: a systematic review. BMC Public Health. 2016;16:173 10.1186/s12889-016-2843-1 . 26895824 PMC4759952

[17] Singkorn O, Apidechkul T, Putsa B, Detpetukyon S, Sunsern R, Thutsanti P, et al Factor associated with alcohol use among Lahu and Akha hill tribe youths, northern Thailand. Subst Abuse Treat Prev Policy. 2019;14(1):5 10.1186/s13011-019-0193-6 . 30678692 PMC6346547

[18] Kumar SG, Premarajan KC, Subitha L, Suguna E, Vinayagamoorthy, Kumar V. Prevalence and pattern of alcohol consumption using Alcohol Use Disorders Identification Test (AUDIT) in Rural Tamil Nadu, India. J Clin Diagn Res. 2013;7(8):1637–9. 10.7860/JCDR/2013/5521.3216 . 24086861 PMC3782918

[19] Supit A, Mamuaja P, Pissu A. Alcohol consumption among college students in Minahasa, Indonesia: a cross-sectional study towards the formulation of intervention strategies. Int J Community Med Public Health. 2017;4(12),4630–4637. 10.18203/2394-6040.ijcmph20175342

[20] Tavolacci MP, Boerg E, Richard L, Meyrignac G, Dechelotte P, Ladner J. Prevalence of binge drinking and associated behaviours among 3286 college students in France. BMC Public Health. 2016;16:178 10.1186/s12889-016-2863-x . 26905284 PMC4765104

[21] Taylor AW, Bewick BM, Makanjuola AB, Qian L, Kirzhanova VV, Alterwain P. Context and culture associated with alcohol use amongst youth in major urban cities: A cross-country population based survey. PLoS One. 2017;12(11):e0187812 10.1371/journal.pone.0187812 . 29155847 PMC5695777

[22] Haug S, Schaub MP, Salis Gross C, John U, Meyer C. Predictors of hazardous drinking, tobacco smoking and physical inactivity in vocational school students. BMC Public Health. 2013;13:475 10.1186/1471-2458-13-475 . 23672294 PMC3658930

[23] Shiferaw D, Kinati T, Fufa G, Assefa L. Prevalence rate of alcohol use and its associated factors among undergraduate students of Jigjiga University. Glob J Addict Rehabil Med. 2017;4(2). 10.19080/GJARM.2017.04.555633

[24] Tesfaye G, Derese A, Hambisa MT. Substance use and associated factors among university students in ethiopia: A cross-sectional study. J Addict. 2014;2014:969837 10.1155/2014/969837 . 24872903 PMC4020400

[25] Carney T, Myers BJ, Louw J, Lombard C, Flisher AJ. The relationship between substance use and delinquency among high-school students in Cape Town, South Africa. J Adolesc. 2013;36(3):447–55. 10.1016/j.adolescence.2013.01.004 . 23453849

[26] Holtes M, Bannink R, Joosten‐van Zwanenburg E, van As E, Raat H, Broeren S. Associations of truancy, perceived school performance, and mental health with alcohol consumption among adolescents. J Sch Health. 2015;85(12):852–860. 10.1111/josh.12341 . 26522174

[27] Goldberg-Looney LD, Sánchez-SanSegundo M, Ferrer-Cascales R, Albaladejo-Blazquez N, Perrin PB. Adolescent alcohol use in Spain: Connections with friends, school, and Other delinquent behaviors. Front Psychol. 2016;7:269 10.3389/fpsyg.2016.00269 . 26973567 PMC4776124

[28] Borsari B, Carey KB. Two Brief Alcohol Interventions for Mandated College Students. Psychol Addict Behav. 2005;19(3):296–302. 10.1037/0893-164X.19.3.296 . 16187809 PMC2663045

[29] Jeffries ER, Lemke AW, Shah SM, Dean KE, Richter AA, Buckner JD. Addictive Behavior Interventions Among College Students. Curr Addict Rep. 2016;3(4):368–377. 10.1007/s40429-016-0117-8 . 28603684 PMC5464746

[30] Jalilian F, Karami Matin B, Ahmadpanah M, Motlagh F, Mahboubi M, Eslami AA. Substance abuse among college students: Investigation the role of hopelessness. Life Sci J. 2014;11(9s):396–399. 10.1177/0972063416651595.

[31] Johannessen EL, Andersson HW, Bjørngaard JH, Pape K. Anxiety and depression symptoms and alcohol use among adolescents—a cross sectional study of Norwegian secondary school students. BMC Public Health. 2017;17(1):494 10.1186/s12889-017-4389-2 . 28535753 PMC5442592

[32] Baines L, Jones A, Christiansen P. Hopelessness and alcohol use: The mediating role of drinking motives and outcome expectancies. Addict Behav Rep. 2016;4:65–69. 10.1016/j.abrep.2016.11.001 . 29511726 PMC5836522

[33] Jaffee WB, D’Zurilla TJ. Personality, problem solving, and adolescent substance use. Behav Ther. 2009;40(1):93–101. 10.1016/j.beth.2008.03.001 . 19187820

[34] Krank M, Stewart SH, O’Connor R, Conrod PJ, Woicik PB, Wall AM. Structural, concurrent, and predictive validity of the substance use risk profile scale in early adolescence. Addict Behav. 2011;36(1–2):37–46. 10.1016/j.addbeh.2010.08.010 . 20826056

[35] Comeau N, Stewart SH, Loba P. The relations of trait anxiety, anxiety sensitivity and sensation seeking to adolescents’ motivations for alcohol, cigarette, and marijuana use. Addict Behav. 2001;26(6):803–25. 10.1016/s0306-4603(01)00238-6 . 11768546

[36] Rounsaville BJ, Kosten TR, Weissman MM, Prusoff B, Pauls D, Anton SF, et al Psychiatric disorders in relatives of probands with opiate addiction. Arch Gen Psychiatry. 1991;48(1):33–42. 10.1001/archpsyc.1991.01810250035004 . 1984760

[37] Wang H, Hu R, Zhong J, Du H, Fiona B, Wang M, et al Binge drinking and associated factors among school students: a cross sectional study in Zhejiang Province, China. BMJ Open. 2018;8:e021077 10.1136/bmjopen-2017-021077 . 29654047 PMC5898305

[38] Oppong Asante K, Kugbey N. Alcohol use by school-going adolescents in Ghana: Prevalence and correlates. Ment Health Prev. 2019;13:75–81. 10.1016/j.mhp.2019.01.009 PMC6716856 31485264

[39] Chen X, Unger JB, Palmer P, Weiner MD, Johnson CA, Wong MM, et al Prior cigarette smoking initiation predicting current alcohol use: Evidence for a gateway drug effect among California adolescents from eleven ethnic groups. Addict Behav. 2002;27(5):799–817. 10.1016/s0306-4603(01)00211-8 . 12201385

[40] Lewinsohn PM, Rohde P, Brown RA. Level of current and past adolescent cigarette smoking as predictors of future substance use disorders in young adulthood. Addiction. 1999;94(6):913–921. 10.1046/j.1360-0443.1999.94691313.x . 10665079

[41] Wicki M, Kuntsche E, Gmel G. Drinking at European universities? A review of students’ alcohol use. Addict Behav. 2010;35(11):913–24. 10.1016/j.addbeh.2010.06.015 . 20624671

[42] Ajilore O, Amialchuk A, Egan K. Alcohol consumption by youth: Peers, parents, or prices? Econ Hum Biol. 2016;23:76–83. 10.1016/j.ehb.2016.07.003 . 27507725

[43] Chauke TM, van der Heever H, Hoque ME. Alcohol use amongst learners in rural high school in South Africa. Afr J Prim Health Care Fam Med. 2015;7(1):755 10.4102/phcfm.v7i1.755 . 26466397 PMC4656918

[44] Romo-Avilés N, Marcos-Marcos J, Marquina-Márquez A, Gil-García E. Intensive alcohol consumption by adolescents in Southern Spain: The importance of friendship. Int J Drug Policy. 2016;31:138–46. 10.1016/j.drugpo.2016.01.014 . 26948500

[45] Haugland SH, Holmen TL, Ravndal E, Bratberg GH. Parental alcohol misuse and hazardous drinking among offspring in a general teenage population: gender-specific findings from the Young-HUNT 3 study. BMC Public Health. 2013;13:1140 10.1186/1471-2458-13-1140 . 24314020 PMC3866523

[46] Murphy E, O’Sullivan I, O’Donovan D, Hope A, Davoren MP. The association between parental attitudes and alcohol consumption and adolescent alcohol consumption in Southern Ireland: a cross-sectional study. BMC Public Health. 2016;16(1):821 10.1186/s12889-016-3504-0 . 27538455 PMC4991068

[47] Ryan SM, Jorm AF, Lubman DI. Parenting factors associated with reduced adolescent alcohol use: a systematic review of longitudinal studies. Aust N Z J Psychiatry. 2010;44(9):774–83. 10.1080/00048674.2010.501759 20815663

[48] Htin K, Howteerakull N, Suwannapong N, TipayamongkholgulI M. Smoking, alcohol consumption and betal-quid chewing among young adult Myanmar laborers in Thailand. Southeast Asian J Trop Med Public Health. 2014;45(4):926–939. https://www.ncbi.nlm.nih.gov/pubmed/25427362 . 25427362

[49] Howteerakul N, Suwannapong N, Than M. Cigarette, alcohol use and physical activity among Myanmar youth workers, Samut Sakhon Province, Thailand. Southeast Asian J Trop Med Public Health. 2005;36(3):790–796. https://www.ncbi.nlm.nih.gov/pubmed/16124457 . 16124457

[50] Saw YM, Saw TN, Yasuoka J, Chan N, Kham NPE, Khine W, et al Gender difference in early initiation of methamphetamine use among current methamphetamine users in Muse, Northern Shan State, Myanmar. Harm Reduct J. 2017;14(1):21 10.1186/s12954-017-0147-0 . 28482847 PMC5422873

[51] Sebena R, Orosova O, Mikolajczyk RT, van Dijk JP. Selected sociodemographic factors and related differences in patterns of alcohol use among university students in Slovakia. BMC Public Health. 2011;11:849 10.1186/1471-2458-11-849 . 22067135 PMC3282827

[52] Lu S, Du S, Hu X, Zou S, Liu W, Ba L, Ma G. Drinking patterns and the association between socio-demographic factors and adolescents’ alcohol use in three metropolises in China. Int J Environ Res Public Health. 2015;12(2):2037–53. 10.3390/ijerph120202037 . 25685952 PMC4344709

